# Modulators of Macrophage Polarization Influence Healing of the Infarcted Myocardium

**DOI:** 10.3390/ijms161226187

**Published:** 2015-12-10

**Authors:** Ellis N. ter Horst, Nazanin Hakimzadeh, Anja M. van der Laan, Paul A. J. Krijnen, Hans W. M. Niessen, Jan J. Piek

**Affiliations:** 1 Department of Pathology, VU University Medical Center, De Boelelaan 1117, Amsterdam 1081 HV, The Netherlands; paj.krijnen@vumc.nl (P.A.J.K.); jwm.niessen@vumc.nl (H.W.M.N.); 2 Department of Cardiology, Academic Medical Center, University of Amsterdam, Meibergdreef 9, Amsterdam 1105 AZ, The Netherlands; a.m.vanderlaan@amc.nl (A.M.L.), j.j.piek@amc.nl (J.J.P.); 3 Interuniversity Cardiology Institute of the Netherlands, Netherlands Heart Institute, Moreelsepark 1, Utrecht 3511 EP, The Netherlands; 4 Institute for Cardiovascular Research, VU University Medical Center, van der Boechorstraat 7, Amsterdam 1081 BT, The Netherlands; 5 Department of Biomedical Engineering and Physics, Academic Medical Center, University of Amsterdam, Meibergdreef 9, Amsterdam 1105 AZ, The Netherlands; n.hakimzadeh@amc.nl; 6 Department of Cardiac Surgery, VU University Medical Center, de Boelelaan 1117, Amsterdam 1081 HV, The Netherlands

**Keywords:** macrophage polarization, myocardial infarction, inflammation

## Abstract

To diminish heart failure development after acute myocardial infarction (AMI), several preclinical studies have focused on influencing the inflammatory processes in the healing response post-AMI. The initial purpose of this healing response is to clear cell debris of the injured cardiac tissue and to eventually resolve inflammation and support scar tissue formation. This is a well-balanced reaction. However, excess inflammation can lead to infarct expansion, adverse ventricular remodeling and thereby propagate heart failure development. Different macrophage subtypes are centrally involved in both the promotion and resolution phase of inflammation. Modulation of macrophage subset polarization has been described to greatly affect the quality and outcome of healing after AMI. Therefore, it is of great interest to reveal the process of macrophage polarization to support the development of therapeutic targets. The current review summarizes (pre)clinical studies that demonstrate essential molecules involved in macrophage polarization that can be modulated and influence cardiac healing after AMI.

## 1. Introduction

Healing of the myocardium post-acute myocardial infarction (AMI) coincides with a general inflammatory response to clear and replace the injured area of the myocardium in the end with scar tissue. Post-AMI, signals from the injured myocardium attract circulating blood monocytes that infiltrate into the myocardium and can differentiate into macrophages [1,2,3]. Two well-established polarized macrophage phenotypes are known, namely the classically-activated M1 macrophages and alternative activated M2 macrophages. M2 macrophage is a collective term to define multiple forms of macrophage activation (M2a, M2b and M2c) that are alternatively activated as compared to M1 macrophages, and they are all involved in type II immune responses, such as the promotion of tissue repair and resolution of inflammation [4]. During the first few days post-AMI, next to neutrophilic granulocytes, classical M1 macrophages dominate the cellular infiltrate and mainly clear cellular debris [5]. Subsequently, these M1 macrophages secrete cytokines, chemokines and growth factors that influence the consequent phases of healing and initiate tissue regeneration coordinated by M2 macrophages (reviewed by [6]). The prolonged presence of M1 macrophages extends the pro-inflammatory environment and causes expansion of the infarcted area in the myocardium post-AMI [7,8]. Then, the formation of scar tissue is hindered by a delayed transition to M2 macrophages predisposing to heart failure development due to adverse remodeling of the injured ventricular wall. Experimental studies that target the inflammatory monocytes or the M1 macrophages post-AMI and thereby diminish the duration of the initial highly inflammatory phase have been shown to improve functional cardiac output post-AMI [7,9,10]. Additionally, studies wherein macrophage polarization towards the M2 phenotype was stimulated have been shown to promote the resolution of inflammation and improve infarct healing post-AMI [11,12]. Therefore, the augmentation of post-AMI healing by modulating the polarization of macrophage subtypes is an appealing concept to beneficially influence healing following AMI [10,11,13]. In the current review, studies that have modulated macrophage polarization are discussed to provide an overview of potential targets to ameliorate the healing process post-AMI.

## 2. Macrophage Polarization

Macrophage polarization is a process that is driven by stimuli of the surrounding (micro) environment, predominantly evoked by pathological processes within organs. The factors driving macrophage polarization include secreted cytokines and growth factors along with microorganism-associated molecular patterns, such as bacterial lipopolysaccharide (LPS) [14]. *In vitro*, M1 macrophage polarization can be induced by the addition of interferon-gamma (IFN-γ), either alone or in combination with other stimulants, including LPS, tumor necrosis factor alpha (TNFα) and granulocyte macrophage colony-stimulating factor (GM-CSF) [4,14]. IFN-γ and LPS are the most widely used to induce M1 macrophage polarization *in vitro*. IFN-γ induces downstream phosphorylation of signal transducers and activators of transcription-1 (STAT1) by Janus kinases (JAK). LPS in particular activates toll like receptor (TLR)-4, which, in turn, can affect the mitogen-activated protein (MAPK) pathway, the interferon regulatory factor (IRF) pathway and the nuclear factor κВ (NF-κВ) pathway by inactivating the NF-κВ kinase inhibitor (IKK)-2. The NF-κВ pathway has also been described to be involved in the regulation of STAT1 activity in M1 macrophages. When NF- κВ activity is diminished through deletion of IKK-2, it is shown in mouse macrophages that STAT1 activity is enhanced [15].This enhanced STAT1 activity contributes in M1 macrophages to the production of nitric-oxide (NO) through inducible NO synthase (iNOS) and the secretion of pro-inflammatory chemokines and cytokines, such as interleukin (IL)-1β, IL-6, IL-12, IL-23 and TNFα, that attract other components of the pro-inflammatory response to eliminate infections [4,16]. The Notch signaling pathway is also involved in LPS-TLR4-induced expression of inflammatory M1 macrophage cytokines. Activation of Notch results in nuclear translocation and binding of the Notch intracellular domain (NCID) to the DNA binding protein immunoglobin kappa J (RBP-J) controlling the expression of IL-12 and iNOS [17].

Polarization of M2 macrophages can be achieved *in vitro* by exposure to IL-4, IL-10, IL-13 or transforming growth factor beta (TGFβ). Both IL-4 and IL-13 signal through the JAK-STAT pathway, leading to the activation of STAT6. Signaling through STAT6 has been shown to be essential for the expression of several M2 macrophage markers [18]. In M2 macrophages, the production of NO is diminished due to the blockage of iNOS, but cytokines, such as TGFβ, IL-10 and the IL-1 receptor antagonist, are produced. The M2 macrophage subtype is involved in the resolution of inflammation and participates in the processes of tissue remodeling and angiogenesis.

Polarized macrophages can theoretically be distinguished by their different receptor expression profiles, as well as differential cytokine production. However, it has to be noticed that also polarized macrophages retain their plasticity to respond to environmental signals [19]. Therefore, a single biochemical marker to discriminate macrophage populations remains difficult, and multiple markers are typically examined to distinguish macrophage phenotypes. In mice, IL-12, major histocompatibility complex (MHC) class II molecules and iNOS2 are considered as M1 macrophage-affiliated markers. However, in humans, iNOS2 induction in M1 macrophages is lacking. In contrast, relevant markers of M2 macrophages in mice include resistin-like-α, arginase 1, chitinase 3-like 3, IL-10 and macrophage mannose receptor 1 (CD206); while human M2 macrophages only display IL-10 and CD206 in the presence of IL-4 [20], but additionally express indoleamine 2,3-dioxygenase [14,20]. To identify human macrophages immunohistochemically in paraffin embedded tissue, the general macrophage population is stained using the CD68 marker. The M2 macrophages are then distinguished from M1 macrophages using an additional CD163 marker [21,22]. However, it has been suggested that the CD163 marker is not M2 macrophage specific and should additionally be identified analyzing CMAF expression, an essential transcription factor for IL-10 [23]. To identify M1 macrophages, phosphorylated STAT1 and recombinant signal binding protein for RBP-J should be added to the CD68 staining.

## 3. Modulators of Macrophage Polarization after AMI

### 3.1. T Regulatory Cells

Important inflammatory cells that are described to influence macrophage polarization are T regulatory cells (Tregs). Tregs are a subset of CD4^+^ T cells that express the forkhead box P3 (FoxP3) transcriptional regulators and are involved in the suppression and regulation of innate immune responses in wound healing following injury [24,25]. Weirater *et al.* [12] described that depletion of circulatory Tregs in mice following AMI induction through permanent coronary ligation resulted in a larger infarcted area of the heart and an impaired resolution of the early inflammatory phase as compared to control-treated AMI mice. Additionally, messenger RNA (mRNA) levels at five days post-AMI in the Tregs depleted mice depicted mainly M1 macrophage-specific markers, indicative of a delayed transition towards the reparative M2 macrophages. Alternatively, expansion of the Tregs post-AMI in mice using the agonistic anti-CD28 antibody showed increased M2 macrophage-associated mRNA levels in the infarcted myocardium at day five post-AMI and increased expression of contributors of myocardial healing, such as osteopontin and M2 macrophage-associated transglutaminase factor XIII [12,26]. Thus, this implicates an important role for Tregs in macrophage polarization and the transition of the highly inflammatory phase towards the healing phase post-AMI.

### 3.2. Interferon Regulatory Factor 5

The transcription factor IRF5 has been demonstrated to be involved in the M1 macrophage polarization. The M1 macrophages namely express IRF5 to a higher degree as compared to M2 macrophages [10,27]. Originally, IRFs were described as regulators of type I interferon expression and signaling. However, it has nowadays been generally established that IRFs also play important roles in the regulation of macrophage and dendritic cell ontogeny [28]. Nine members of the mammalian IRF family have been described, and each member has been associated with a specific function [28]. Activation of IRF5 has been shown to play an essential role in macrophage plasticity, resulting in both an increased expression of M1 macrophage markers, as well as hindering the expression of M2 macrophage markers [10,11,27]. Courties *et al.* [10] showed in ApoE^−/−^ mice that at four days post-AMI, infiltrated macrophages in the heart highly express IRF5, after which levels decreased drastically around eight days post-AMI. This confirmed the hypothesis that M1 macrophages dominate the infarct area early after AMI and diminish in number afterwards. *In vivo* silencing of IRF5 expression in these mice, using intravenous administration of siRNA-encapsulated nanoparticles, resulted in decreased expression levels of M1 macrophage-specific genes, including TNFα and IL-1β in the heart at four days post-AMI. The expression of M2-related genes, on the other hand, was not affected [10]. These results indicate that silencing IRF5 attenuates M1 macrophage polarization and can thereby support the resolution of inflammation and scar tissue formation performed by M2 macrophages. Remarkably, since the level of M2 macrophage-related genes remained unaffected, it seems unlikely that the M1 macrophages are ”switched” towards M2 macrophages [10]. Thus, how IRF5 modulates macrophage polarization remains to be unraveled. Additionally, using cardiac magnetic resonance imaging (CMR), Courties *et al.* showed that although infarct size was not different between the si-IRF5 treatment group and the control group at Day 1 post-AMI, the si-IRF5 treatment group developed less left ventricular dilatation three weeks post-AMI as compared to the control group. The authors suggest that since si-IRF5 was only administrated during the first five days post-AMI, the difference in post-AMI ventricular dilatation development is most likely the result of improved infarct healing in si-IRF5-treated mice [10]. Albeit, no tissue analysis of the infarct area at three weeks post-AMI was included in this study to confirm this hypothesis.

### 3.3. Collapsin Response Mediator Protein-2

The collapsin response mediator protein-2 (CRMP-2) coincides with IRF5 activation and has been suggested as a novel protein involved in macrophage polarization [11,12]. *In vitro* studies revealed that expression of CRMP-2 is higher in M1 macrophages as compared to M2 macrophages [11]. Moreover, silencing of CRMP-2 expression in cultured M1 macrophages resulted in a switch towards a M2 macrophage gene expression profile, namely a reduction of TNFα and CD86 expression and an increase in IL-10 expression. Zhou *et al.* [11] silenced the expression of CRMP-2 using nanoparticles (RNAi) in ApoE^−/−^ mice that underwent permanent coronary artery ligation. Silencing of CRMP-2 did not change the total amount of infiltrated macrophages in the first 10 days post-AMI. However, in the RNAi-treated mice, expression of the M1 macrophage marker CD86 was lower, whereas expression of the M2 macrophage-like marker CD206 was higher, as compared to the control ApoE^−/−^ AMI mice. This is indicative of a switch towards the M2 macrophages post-AMI [11]. Conversely, mRNA level analysis in the infarcted myocardium showed that the number of inflammatory cells, such as monocytes, neutrophils and macrophages, was lower in CRMP-2 RNAi AMI mice as compared to the control RNAi at Day 7 and Day 28 post-AMI. Moreover, the extent of myocardial fibrosis was lowered in the CRMP-2 knockdown ApoE^−/−^ AMI mice, which most likely contributed to an enhanced recovery of cardiac function during 28 days post-AMI [11]. The exact mechanism that connects CRMP-2 with IRF5 remains to be elucidated, although it was suggested that CRMP-2 is involved in the transcriptional regulation of IRF5 [11].

### 3.4. Suppressors of Cytokine Signaling

Another transcription factor family that has been shown to modulate macrophage polarization is the suppressors of cytokine signaling (SOCS). SOCS are target genes of the Janus-associated kinase (JAK)/STAT pathways performing a negative feedback loop, which eventually inhibits the propagation of cytokine secretion. Amongst the SOCS isoforms, SOCS1 and SOCS3 are most widely described in the regulation of macrophage polarization [29,30,31,32]. Their expression in resting macrophages is generally low, but increases upon macrophage activation. It has been shown that a high SOCS3 expression is associated with M1 macrophages, whereas an increase in the SOCS1/SOC3 ratio could be a potential marker for M2 macrophages [29,31,32]. Silencing SOCS1 in macrophages both *in vitro* and *in vivo* resulted in an enhanced expression of pro-inflammatory cytokines, such IL-6, IL-12 and TNFα [32,33]. This suggests that SOCS1 normally sustains the expression of these M1 macrophage cytokines and thereby regulates the intensity of their inflammatory reaction. In the absence of SOCS3, human and mouse macrophages show reduced expression of M1 macrophage-related cytokines, such as IL-1β, IL-6 and IL-23 after LPS/IFN-γ stimulation [29,30]. Moreover, the responsiveness to IL-4 was restored, which is normally inhibited in M1 macrophages. This coincided with an immune-regulatory phenotype, such as an increased expression of arginase and CD206 and an attenuated increase of NO after IFN-γ/LPS stimulation [29,30]. In mice, deletion of SOCS3 preceding permanent AMI using SOCS3-flox mice has been shown to reduce the progression of adverse remodeling post-AMI [34]. At Day 2 post-AMI, mRNA levels of M1-associated genes, such as IL-6 and GM-CSF, were namely lower in the myocardium of SOCS3 knockout mice as compared to control mice. However, the expression of macrophage-specific markers was not studied [34].

In conclusion, although SOCS1 and SOCS3 expression in macrophages has been related to macrophage polarization, the exact role of SOCS on macrophage polarization during healing post-AMI is still unknown.

## 4. Targeting Monocytes in Clinical Studies

**Figure 1:**
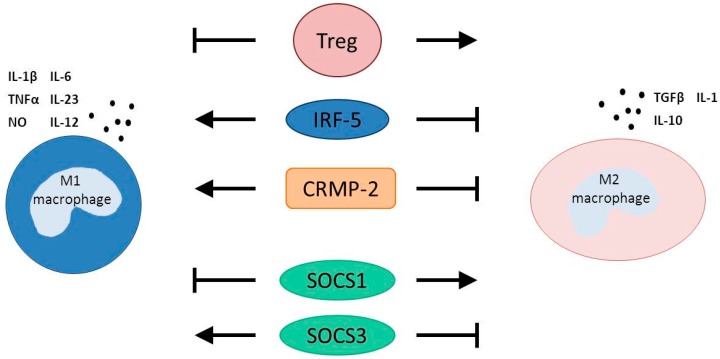
Essential modulators of macrophage polarization. Preclinical studies have demonstrated that an increased activity of IRF5, CRMP-2 and SOCS3 in macrophages reduces the polarization towards M2 macrophages (block arrows) and stimulates M1 macrophage polarization (point arrows). Tregs and SOCS1 have been implicated in the polarization of M2 macrophages (point arrows), while repressing M1 macrophage polarization (block arrows). For therapeutic clinical interventions to ameliorate healing post-myocardial infarction, it is of interest to either stimulate the factors that stimulate M2 macrophage polarization or inhibit the factors that stimulate M1 macrophage activity. Treg = T regulatory cells; IRF5 = interferon regulatory factor 5; CRMP-2 = collapsin response mediator-2; SOCS = stimulators of cytokine signaling. From the cytokines: IL = interleukin; TNFα = tumor necrosis factor alpha; NO = nitric oxide; TGFβ = transforming growth factor beta.

In the previous sections, it was discussed that a switch or stimulation towards the M2-like macrophages post-AMI results in an improvement of cardiac function recovery post-AMI, at least in pre-clinical animal studies (Figure 1).

In line with this, in clinical studies, it was shown that high levels of pro-inflammatory monocytes in the circulation of patients with AMI displayed a poor prognostic outcome [5,35]. This is of interest, as circulating monocytes contribute to macrophage accumulation in injured myocardium.

Monocytes originate from progenitors cells in the bone marrow and are released into the peripheral blood, where they circulate before they are recruited into tissues [36]. In the peripheral blood, human monocyte populations are distinguished into subgroups mostly based on their expression of cell surface receptor markers CD14 and CD16. Classical monocytes are defined as CD14^++^CD16^−^, while intermediate monocytes are identified as CD14^++^CD16^+^ and non-classical monocytes are classified as CD14^+^CD16^+^ [37]. The classical monocytes are mostly described to be involved in proteolytic, phagocytic and highly inflammatory processes, whereas the intermediate monocytes are shown to be involved in promoting angiogenesis and the non-classical monocytes to promote healing and attenuate inflammatory processes [38]. It remains disputable how monocyte subsets relate to the differentiation of macrophage subsets once they enter the perivascular space. It has been described in mice that following accumulation of classical monocytes in inflammatory sites, this subset often differentiates into M1-like macrophages [39]. However, it has also been demonstrated in mice that following recruitment of classical monocytes into tissue, these classical monocytes can switch their phenotype to obtain an anti-inflammatory profile and eventually differentiate into M2-like macrophages or even retain their monocyte phenotype [5,8,40]. Theoretically, modulation of monocyte mobilization in clinical settings could have therapeutic potential by reducing pro-inflammatory blood monocyte and macrophage tissue infiltration. In mice, it has been demonstrated that reduced recruitment of the classical monocytes post-AMI by targeting the classical monocyte recruitment chemokine C–C motif receptor 2 (CCR2) reduces myocardial infarct size, attenuates infarct inflammation and limits adverse left ventricular remodeling [7,41]. However, no clinical trials have been performed yet in human patients that successfully modulate the pro-inflammatory cytokine pathway post-AMI. Ultimately, imaging the potential differentiation of monocytes and macrophages in patients could potentially have prognostic effects. However, it is at the same time valuable in testing the therapeutic efficacy of clinical interventions aimed at modulating the activity and infiltration of monocyte and macrophages subsets in atherosclerotic plaques or infarcted tissue post-AMI.

## 5. Conclusions

The current review emphasizes the essence of macrophages during healing following AMI. Modulating the presence of the M2 macrophages during the inflammatory response post-AMI has been demonstrated in several studies to ameliorate infarct healing and diminish the development of adverse cardiac remodeling post-AMI. Macrophage polarization is dependent on multiple processes, such as interaction with other leukocytes and systemic factors, but it remains uncertain whether these are connected to each other. It has clearly been shown that Tregs can stimulate M2 macrophage polarization, whereas CRMP-2 and IRF5 stimulate M1 macrophage polarization. Additionally, stimulating Tregs or inhibiting CRMP-2 or IRF5 eventually results in a reduction of adverse cardiac remodeling, at least in preclinical animal studies (Figure 1). It would therefore be of great interest to unravel therapeutic strategies to influence these factors in clinical settings. The use of loaded nanoparticles appears to be a potential therapeutic strategy to target specific cell types following the onset of diseases (reviewed in [42]). Additionally, the use of RNAi in the nanoparticles is widely regarded to be a promising treatment technology to influence particular cell types, such as monocytes and macrophages [11,43]. Loading the nanoparticles with RNAi that either reduces the classical monocytes and infiltration of M1 macrophages or stimulates the non-classical monocytes and M2 macrophage infiltration should be a potential aim for clinical studies to improve cardiac function post-AMI; for example, silencing CCR2 to reduce peripheral classical monocytes or silencing CRMP-2 to favor the infiltration of M2 macrophages post-AMI [7,11,41]. However, the focus on clinical studies has nowadays mainly been on targeting the monocyte subsets as precursors of the macrophages to influence macrophage polarization post-AMI. However, imaging of monocyte and macrophage subsets in the clinical setting would be of great interest to use as prognostic factors, to test therapeutic efficiencies and to determine the fate of monocyte subsets once infiltrated into the myocardium.
